# Insight into the liposomal encapsulation of mono and bis-naphthalimides†

**DOI:** 10.1039/d3pm00060e

**Published:** 2024-03-08

**Authors:** Abdullahi Magaji Dauda, Thomas Swift, Richard Telford, Hend A. A. Abd El-wahab, Chhanda Charan Danta, Klaus Pors, Amalia Ruiz

**Affiliations:** a Institute of Cancer Therapeutics, School of Pharmacy and Medical Sciences, Faculty of Life Sciences, University of Bradford Bradford UK g.ruizestrada@bradford.ac.uk; b School of Chemistry and Biosciences, Faculty of Life Sciences, University of Bradford Bradford UK

## Abstract

Mitonafide-loaded liposomes are a promising strategy to overcome the neurotoxicity observed in clinical trials for this drug. This study investigates the influence of loaded mitonafide or a dimer analogue on different liposomal formulations and their therapeutic efficacy *in vitro*. Physicochemical properties of the liposomes were manipulated using different loading methods (namely bilayer or core loading) and varying the rigidity of the bilayer using distinct phospholipid compositions. Our results demonstrated that the mitonafide dimer analogue had a comparable encapsulation efficiency (EE%) into the liposomes when loaded into rigid or flexible bilayers in contrast to the low mitonafide monomer EE%. A pH gradient core loading method resulted in a more efficient mechanism to load the monomer into the liposomes. DOSY NMR and spectrofluorometric studies revealed key differences in the structure of the vesicles and the arrangement of the monomer or the dimer in the bilayer or the core of the liposomes. The *in vitro* assessment of the formulations using MDA-MB-231 and RT-112 cells revealed that a flexible lipid bilayer allows a faster drug release, which correlated well with the spectroscopy studies. This study investigated for the first time that the characteristics of the lipid bilayer and the loading method influence the encapsulation efficacy, colloidal properties, photoactivity and stability of mono and bis-naphthalimides loaded in a liposomal carrier, essential factors that will impact the performance of the formulation in a biological scenario.

## Abstract 2

The characteristics of the lipid bilayer and the loading method influence the encapsulation efficacy, colloidal properties, photoactivity and stability of mono and bis-naphthalimides loaded in a liposomal carrier.

## Graphical abstract

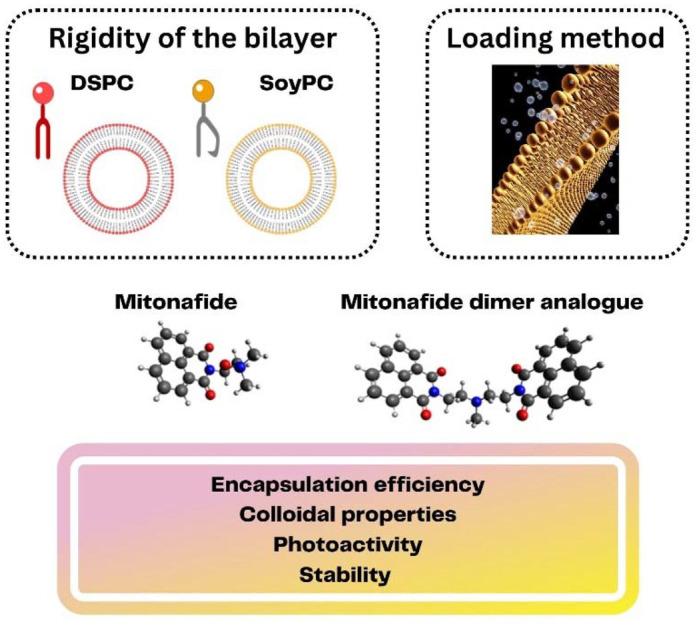

## Introduction

Naphthalimide derivates have a long history in Medicinal Chemistry due to their photochemical and spectroscopic properties and high antitumour activity. In 1973, Braña *et al.* developed the first class of naphthalimides and described their biological activity.^1^ Since then, the development of functional 1,8-naphthalimide derivates as DNA intercalators, anticancer and bioimaging agents has resulted in several candidates entering clinical trials.^2^ Two leading compounds of this group, amonafide and mitonafide, have entered into phase II clinical trials. Amonafide has been evaluated for prostate cancer (NCT00087854) and poor-risk acute leukaemia,^3–5^ whilst the antitumoural activity of mitonafide has been investigated in phase I and II clinical trials for advanced and relapsed colorectal cancer and non-small cell lung cancer.^6,7^ Mitonafide was administered in a short schedule (at doses from 15.4 to 138.6 mg m^−2^ during five days), showing promising results inhibiting tumour growth. However, the study was interrupted due to the appearance of neurotoxicity in different patients.^8^ A slower administration of mitonafide reduced the adverse effects on the central nervous system, but this strategy lacked efficacy in inhibiting tumour growth.^7^ Similar results were obtained for amonafide, where myelosuppression halted the studies.^9,10^

A way to improve DNA binding affinity and the antitumour effect of naphthalimides is to synthesise two subunits of 1,8-naphthalimide connected by a linker or spacer. Thorough research has been done studying the influence of the linker or the molecule's symmetry in the biological activity of these dimers.^11^ Candidates like elinafide, developed by Braña *et al.*, or DMP 840, synthesised by Chen and co-workers, have exhibited excellent tumour inhibition in different preclinical studies.^12–14^ Despite the significant results in the cellular potency of the dimers, problems of poor solubility and cumbersome synthesis of the dimers have been described by Wu *et al.*^15^

Liposomes are a well-established family of nanoparticles used to deliver chemotherapeutic and antifungal drugs, genes, vaccines, and imaging agents.^16^ A meta-analysis of 14 oncological clinical trials has shown that despite the efficacy in patients being the same between liposomal and conventional chemotherapy, the liposomal formulations enhanced tolerability by changing the side effect profile.^17^ These lipid-based nanocarriers have a significant advantage in terms of biocompatibility, biodegradability and reduced toxicity. Additionally, since the drug can be encapsulated in the internal aqueous core or the phospholipid bilayer, they offer a versatile technological platform for drug delivery.

Few individual studies have reported the encapsulation of naphthalimides in liposomal formulations. For instance, Gao *et al.* have studied three different cationic lipids with a naphthalimide moiety to enhance gene transfection efficiency.^18^ Others have reported the conjugation of a naphthalimide to a cationic peptide (BP100) that is active against bacteria and displays low toxicity towards eukaryotic cells. In this article, the authors investigated the permeabilising capacity of the peptide by changing the lipid composition of the liposomal bilayer by monitoring the fluorescence properties of naphthalimides.^19^ Similarly, the fluorescence properties of naphthalimides have been explored to develop self-reporting vectors for nucleic acid delivery using a liposomal carrier.^20,21^ To the best of our knowledge, only one article has been published studying the loading of a naphthalene-based diimide derivative (AN169) in liposomes avoiding the use of DMSO as a solubilising agent in *in vitro* and *in vivo* experiments.^22^ Encouragingly, the authors developed a sterically stabilised formulation with the desired size and polydispersity index for intravenous administration and preserved drug loading after lyophilisation. The present study aims to investigate the loading of mono and bis-naphthalimide compounds in liposomes to improve their solubility and future biomedical applications. Herein, we have successfully demonstrated the preparation of PEGylated liposomal formulations with the desired size (<200 nm) and dispersity index (<0.25) encapsulating mitonafide and a dimer analogue, respectively. We systematically studied the effect of the lipid bilayer and the loading method to maximise the encapsulation efficiency of the monomer or the dimer. Our work has highlighted, for the first time, the influence of the structural characteristics of the liposomes on the overall fluorescence, stability and *in vitro* efficacy of mono and bis-naphthalimides formulations for cancer therapy.

## Materials and methods

### Materials

All solvents used were purchased from Merck (methanol (34860), chloroform (366927), dimethyl sulfoxide (DMSO) (1.16743)). Chemicals for naphthalimide target compounds were purchased from Merck, including 1,8-naphthalic anhydride (N1607), 3-nitro-1,8-naphthalic anhydride (N19001), *N*,*N*-bis(3-aminopropyl)methylamine (690228) and *N*,*N*-dimethylethylenediamine (D158003). Soybean phosphatidylcholine (Lipoid S100 97281-47-5), 1,2-distearoyl-*sn-glycero*-3-phosphocholine (DSPC, Lipoid, 816-94-4), 1,2-distearoyl-*sn-glycero*-3-phosphoethanolamine-*N*-[methoxy(polyethylene glycol)-2000] (DSPE-PEG_2000_ Lipoid 147867-65-0), were generous gifts from Lipoid GmbH (Ludwigshafen, Germany). Cholesterol (Merck C8667) sodium chloride (Merck, 71383), HEPES sodium salt (Merck, 75277-39-3), d-mannitol (Merck, M9647), resazurin sodium salt (Merck, R7017) and sterile 0.2 μm pore size syringe filters were purchased from Thermo Fisher Scientific (UK). Roswell Park Memorial Institute (RPMI) 1640 medium, Dulbecco's PBS (1×), penicillin–streptomycin solution liquid (10000 units per mL), glutamine supplement 200 mM, 0.25% trypsin/EDTA, were purchased from ThermoFisher Scientific (UK). Foetal Bovine Serum (FBS), heat-inactivated (F0804-500ML) was obtained from Merck (UK).

### Methods

#### Synthesis of mono and bis-naphthalimides

##### N-(2-(Dimethylamino)ethyl)-3-nitronaphthalimide (mitonafide)

3-Nitro-1,8-naphthalic anhydride (0.3 g, 1.23 mmol, 1 eq.) was dissolved in 5 mL CH_2_Cl_2_ and CH_3_OH (4 : 1 v/v) and *N*,*N*-dimethylethylenediamine (0.15 ml, 1.35 mmol, 1.1 eq.) was added. The resulting solution was stirred at room temperature (RT) for 1 hour before the solvent was removed *in vacuo* (ESI Scheme S1†). The crude compound was purified by flash column chromatography using CH_2_Cl_2_–CH_3_OH (95 : 5 v/v) as eluent to afford a yellowish cream product (0.28 g, 72% yield). ^1^H-NMR (400 MHz, CDCl_3_) ppm: 9.33 (d, *J* = 2.4 Hz, 1H, ArH), 9.14 (d, *J* = 2.4 Hz, 1H, ArH), 8.79 (dd, *J* = 1.2, 7.6 Hz, 1H, ArH), 8.43 (m, 1H, ArH), 7.96 (m, 1H, ArH), 4.37 (t, *J* = 6.8 Hz, 2H, CONC*H*_2_), 2.69 (t, *J* = 6.8 Hz, 2H, CONCH_2_C*H*_2_), 2.36 (s, 6H, CH_3_). ^13^C-NMR (100 MHz, CDCl_3_) ppm: 163.27, 162.41, 146.38, 135.63, 134.22, 132.57, 129.99, 129.13, 124.66, 124.11, 122.94, 56.96, 45.98, 38.70. LCMS: 314.31 (M + H)^+^, 100%, 336.31 (M + Na)^+^, 269.20 (M − N(CH_3_)_2_); UV: 271.97 & 333.97 nm.

##### 2,2′-((Methylazanediyl)bis(propane-3,1-diyl))bis(1H-benzo[de]isoquinoline-1,3(2H)-dione) (mitonafide analogue dimer)

A solution of 1,8-naphthalic anhydride (2.0 eq., 2.72 g) was prepared in ethanol, and *N*,*N*-bis(3-aminopropyl)methylamine (1.0 eq., 1.1 ml) was added before stirring at 78 °C for 4 hours. After the reaction was completed, the reaction mixture was washed with ice-cold NaHCO_3_ and ice-cold brine, dried over MgSO_4_, filtered, and the organic phase evaporated *in vacuo* (ESI Scheme S1†). The residue was purified by column chromatography (silica gel, eluent mixture: CH_2_Cl_2_–CH_3_OH (98 : 2 v/v), affording the titled compound (2.8 g, 82% yield)). ^1^H-NMR (DMSO-d6) *δ* (ppm): 1.75 (m, 4H), 2.30 (s, 3H), 2.40 (t, 4H), 4.10 (t, 4H), 7.70 (dd, 4H), 8.30 (d, 4H), 8.40 (d, 4H). ^13^C-NMR (DMSO-d6) *δ* (ppm): 26.20, 47.50, 57.20, 123.50, 125.70, 128.30, 129.50, 138.20, 159.80. LCMS: 506 (M + H)^+^, 100%.

### Liposome preparation

Phospholipids were dissolved in a mixture of chloroform and methanol (4 : 1 v/v) and placed in a 25 mL round-bottom flask. Liposomes were prepared using the lipid film hydration method. Briefly, the organic solvents were evaporated under reduced pressure at 60 °C for 1 hour using a rotary evaporator (BÜCHI, Labortechnik AG) and then flushed with an N_2_ stream to remove any residual traces of organic solvent. To study the effect of the rigidity of the liposomal bilayer, two phospholipids with different melting points were selected, soybean phosphatidylcholine (SoyPC) or 1,2-distearoyl-*sn-glycero*-3-phosphocholine (DSPC) as the main phospholipid component of the lipid film. To achieve a final lipid concentration of 5 mM, the dried lipid films of SoyPC or DSPC (SoyPC or DSPC/Chol/DSPE-PEG_2000_ 95/50/5 molar ratio) were hydrated with one of the following buffers: ammonium sulphate pH 5.4 (240 mM (NH_4_)_2_SO_4_), HBS (20 mM HEPES and 0.8 wt% NaCl, pH 8.5) or mannitol 5%. Following hydration at 60 °C for 30 minutes, liposomes were downsized by sonication using a probe sonicator (Sonics VC505) through 3 cycles of 30 s on & 10 s off, 40–41% amplitude. After sonication, liposomes were allowed to anneal for a minimum of 2 hours at RT.

### Drug loading

#### Bilayer loading

To load the drugs in the bilayer of the liposomes, mitonafide, monomer or dimer forms, were dissolved in the mixture of chloroform and methanol (4 : 1 v/v) together with the lipids at a 20 : 1 lipid : drug weight ratio and followed the liposomes preparation procedure described above. For bilayer-loaded liposomes, an HBS buffer (20 mM HEPES and 0.8 wt% NaCl, pH 8.5) was used for hydration to obtain a final lipid concentration of 5 mM.

#### Core loading

To investigate the loading of mitonafide (monomer) in the core of the liposomes, two different methods were studied. For the passive loading method, the drug was dissolved in mannitol 5%, and this solution was used to hydrate the lipid films at a 20 : 1 lipid : drug weight ratio, as described above. Following hydration at 60 °C for 30 minutes, liposomes were downsised by sonication, and they were allowed to anneal for a minimum of 2 hours at RT. To prepare the remotely loaded liposomes, lipid films were hydrated with ammonium sulphate pH 5.4 (240 mM (NH_4_)_2_SO_4_). After downsising the liposomes by sonication, (NH_4_)_2_SO_4_ buffer was exchanged with HBS (20 mM HEPES and 0.8 wt% NaCl, pH 8.5) using a Sephadex™ G-25 PD-10 column (Cytiva, Fisher, UK). After buffer exchange, liposomes were incubated with the drug at a 20 : 1 lipid : drug weight ratio at 60 °C for 30 min. Following incubation, liposomes were allowed to anneal for at least 2 hours at RT. The unencapsulated drug was removed using a PD-10 column. Drug encapsulation efficiency (EE%) was determined *via* HPLC.

### Chemical analysis

#### Dynamic light scattering and zeta potential

Colloidal properties of bilayer-loaded liposomes were determined by measuring their hydrodynamic size, PDI and ζ-potential (ZP) using a Zetasizer NanoZS90 (Malvern, UK). Size and ZP measurements were performed using disposable polystyrene cells and plain folded capillary zeta cells (Malvern, UK). Liposome samples were diluted 10-fold in deionised water for size or 100-fold for ZP measurements. All measurements were performed at 25 °C. Size measurements were performed with 3 measurements, each with 15 scans, while ZP measurements were performed with 6 measurements, each with 20 scans.

#### UV/Vis characterisation

Absorbance measurements of solutions were carried out by studying dilute solutions of the mono and bis naphthalene compounds in chloroform. Solutions were placed in 1 cm quartz cuvettes and measured using a Cary 50 Varian probe UV-visible spectrophotometer. Spectra were recorded by taking the average of 5 scans for each sample within the 200 to 400 nm range. Cary WinUV scan application version 02.00(25) software was used to archive and export the spectra.

#### High-performance liquid chromatography (HPLC) analysis

Quantification of drug-loaded liposomes was performed by solubilising the purified liposomes in methanol for HPLC analysis. Quantification of the drugs was assessed using the corresponding calibration curve. Drug EE% was determined by taking the ratio between the concentration of the encapsulated drug after purification using a PD-10 column and the initial drug concentration.
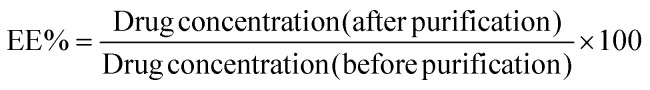


Calibration curves with mitonafide (monomer and dimer) were generated using different concentrations in the range of 5–50 μM in methanol. These compounds were dissolved in methanol before injection into the HPLC machine. A mobile phase gradient was prepared for the sample injection and run as described in the ESI (Table 2 and Fig. 8†). Mobile phase A is constituted of 90% deionised water (dH_2_0), 10% HPLC grade methanol, and 1% formic acid (FA), mobile phase B consists of 90% HPLC grade methanol, 10% dH_2_0 and 1% FA, and mobile phase C was 50/50 A and B, and which served as a wash for the HPLC between runs. All mobile phases were filtered with a hydrophilic 0.45 μM nylon membrane (47 mm thick). The time gradient for the mobile phase is described in the ESI.† Samples were injected every 35 minutes with an injection volume of 10 μL.

#### Inductively coupled plasma mass spectrometry (ICP-MS)

In order to evaluate the titanium content of the formulations samples were sent for analysis to Analytical Innovations©, Wheatley Park, Mirfield, West Yorkshire, United Kingdom. See details in ESI.†

#### Nuclear magnetic resonance

NMR analysis of solutions was carried out on a Bruker Avance Neo 600 MHz spectrometer equipped with an iProbe sensor. Liposomes were prepared at a final lipid concentration of 5 mM as described above and loaded with mitonafide (monomer or dimer) in the core or the bilayer. After preparing the various formulations, liposomes were concentrated using a Vivaspin® concentrator, 50 kDa MWCO (ThermoFisher, UK) to a final volume of 300 μL. Then samples were diluted in 1 mL of deuterium oxide (D, 99.9%) (Cambridge Isotope Laboratories Inc., USA). Diffusion NMR measurements were carried out over 64 gradient steps to determine the diffusion of both the solvent ^1^H and liposome PEG ^1^H. These were used in combination to produce an apparent *R*_H_ of PEG chain flexibility using Stokes–Einstein equations as detailed by Swift *et al.*^23^ NMR of polymer chains linked to nanoparticles is complex, and separation between any free PEG and fully immobilised PEG may be impossible.

#### Fluorescence analysis

Emission measurements were performed on a HORIBA Fluoromax-4 with time-correlated single photon counting (TCSPC) accessories. Samples were diluted in HBS buffer (20 mM HEPES and 0.8 wt% NaCl, pH 8.5) or mannitol 5% depending on their preparation method at a final drug concentration of 2 μM, and measured using 1 cm path length quartz cuvettes to determine their fluorescence properties. A solution of these compounds was prepared in DMSO at the same concentration for comparison with the free monomer or dimer. Excitation was carried out at multiple wavelengths, and the emission was recorded up to 800 nm. Fluorescence lifetime measurements were carried out at 340 nm excitation and 380 nm emission. Dual exponential decays of fluorescence emission were fitted using HORIBA DAS6 software and reduced to an average fluorescent excited state lifetime (see ESI†).

### Computational modelling studies

Calculations on molecular structures were carried out comparing the molecular structure of the monomer and dimer under investigation. Each molecule was initially structurally optimised by DFT methods (PBE-D3/TZV(2d) non-H atoms, TZV(p) H atoms). The absorption spectra were predicted using time-dependent density functional theory (TD-DFT) with a CPCM solvation model of water. All calculations were carried out in the program orca.^24^

### *In vitro* studies

#### Cell culture

Human breast adenocarcinoma cells (MDA-MB-231) and urinary bladder transitional cell carcinoma (RT-112) cells were obtained from American Type Culture Collection (ATCC, USA). Cells were cultured in RPMI 1640 (Invitrogen Gibco Life Technologies), supplemented with 10% heat-inactivated FBS, 50 U mL^−1^ penicillin, 50 μg mL^−1^ streptomycin, and 1% l-glutamine and maintained in a humidified incubator at 37 °C and 5% CO_2_.

#### Cytotoxicity

Both cell lines were trypsinised, and cells were stained with trypan blue (0.4%, 1 : 1 v/v ratio) and counted using a haemocytometer. Cells were seeded (1.7 × 10^4^ cells per well, 200 μL per well) in 96-well culture plates in complete RPMI 1640 media. The next day, 6.25 μM of mitonafide (monomer or dimer)-loaded liposomes were prepared in RPMI full media and added to each well. Cells were incubated at 37 °C for 24 h, and cell viability was assessed using a resazurin assay. Cells were incubated with 0.01 mg mL^−1^ resazurin solution for 4 h. After incubation, the media were collected and transferred to a clean 96-well plate and the fluorescence (*λ*_ex_ = 544 nm, *λ*_em_ = 590 nm) was read using a Fluoroskan (ThermoFisher, UK) plate reader. Six replicates per condition were used. The results were expressed as the percentage of cell viability (mean ± SD) and normalised to control untreated cells.

## Results and discussion

### Synthesis and characterisation of mono and bis-naphthalimides

**Fig. 1:**
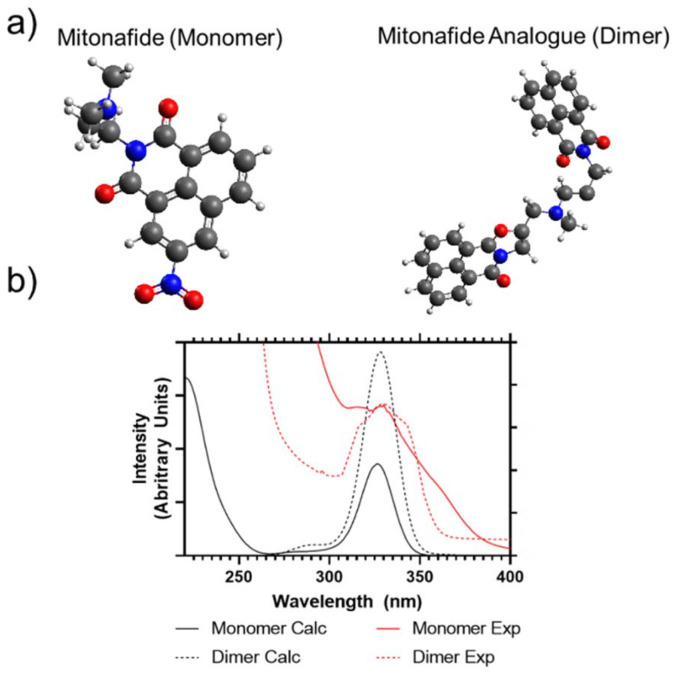
(a) Gas optimised structure predictions of mitonafide monomer (left) and analogue dimer (right) used for UV/Vis absorption calculations. (b) Calculated absorption spectrum of monomer and dimer electronic structures (220–400 nm range) compared to experimentally measured values of samples.

The mitonafide monomer and dimer analogue are characterised by the presence of the typical DNA intercalating tricyclic ring system based on the napthalimide pharmacophore. In the case of the dimer, they are connected by a methylamino propyl diamine linker. The alkyl diamine struture is present in both the monomer and dimeric compound. However, the alkyl group is lengthened from two carbons to three, further separating the extant amine from the conjugated aromatic centre. The synthesis of both compounds produced reasonable yields (72 and 82%, respectively) after the products were purified by flash chromatography. The compounds were characterised by ^1^H, ^13^C NMR to confirm their identity and purity. The mitonafide monomer and dimer were analysed to determine their solution properties. UV/Vis absorption spectra were predicted for the monomer and dimer using computational calculations (ESI Fig. 1†) and compared to experimental results (Fig. 1a,b). This showed that the electronic properties of both compounds following photon absorption at approximately 340 nm are similar – but the two naphthalimide groups in the dimer could exhibit combined or independent frontier molecular orbital transitions.

Most of the compounds having this heteroaromatic structure are photoluminescent. The monomer and the dimer were analysed by fluorescence spectroscopy, and whilst we have predicted from UV/Vis analysis that the peak absorption wavelength would be 340 nm, an improved efficiency of emission intensity was observed following 370 nm excitation. Both compounds showed broad photoluminescence emission from 380 to 600 nm when analysed in DMSO. However, the monomer had a broader, multimodal emission compared to the dimer, which focused more on the primary emission peak of 420 nm (ESI Fig. 2†). TCSPC was also carried out to determine their fluorescence lifetimes, as shown in ESI Fig. 3.† The lifetime of the monomer could be well modelled using a single exponential decay (Chisq = 1.18), whilst the dimer could not be fitted to a single exponential decay (Chisq = 6.72). So, only dual exponential decays are reported from this study (Chisq = 1.66). The average apparent lifetimes (*τ*) were 17.2 ± 0.13 ns for the monomer and 13.3 ± 0.61 for the dimer, respectively.

### Mono and bis-naphthalimides loading in liposomes using different methods

**Fig. 2:**
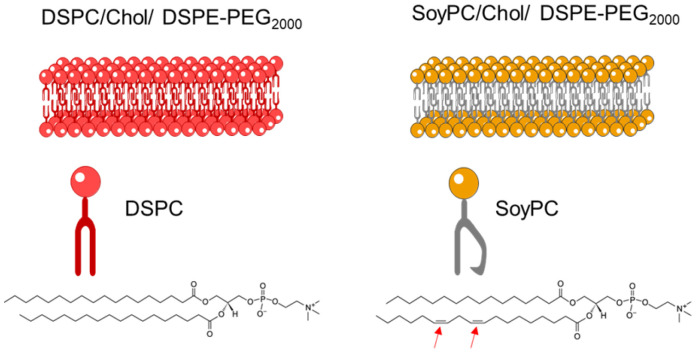
Schematic representation of the liposomal bilayer of two formulations in the study: (left) DSPC or (right) SoyPC/Chol/DSPE-PEG_2000_ (molar ratio: 95/50/5).

Liposomes were prepared using the thin lipid film hydration followed by the sonication method. Since the characteristics of the bilayer highly influence the colloidal properties and loading capacity of the liposomes, two different formulations were studied (SoyPC or DSPC : Chol : DSPE-PEG, (95/50/5 molar ratio)). As illustrated in Fig. 2, DSPC is a phospholipid with two 18 C saturated chains. This structure enhances dispersion attraction force between chains, resulting in a tighter bilayer packing. Meanwhile, SoyPC is a phospholipid that contains two unsaturations in one of the fatty acid chains. These *cis* double bonds have prohibited chain rotation and disrupted chain packing, resulting in higher membrane fluidity.^25^

**Fig. 3:**
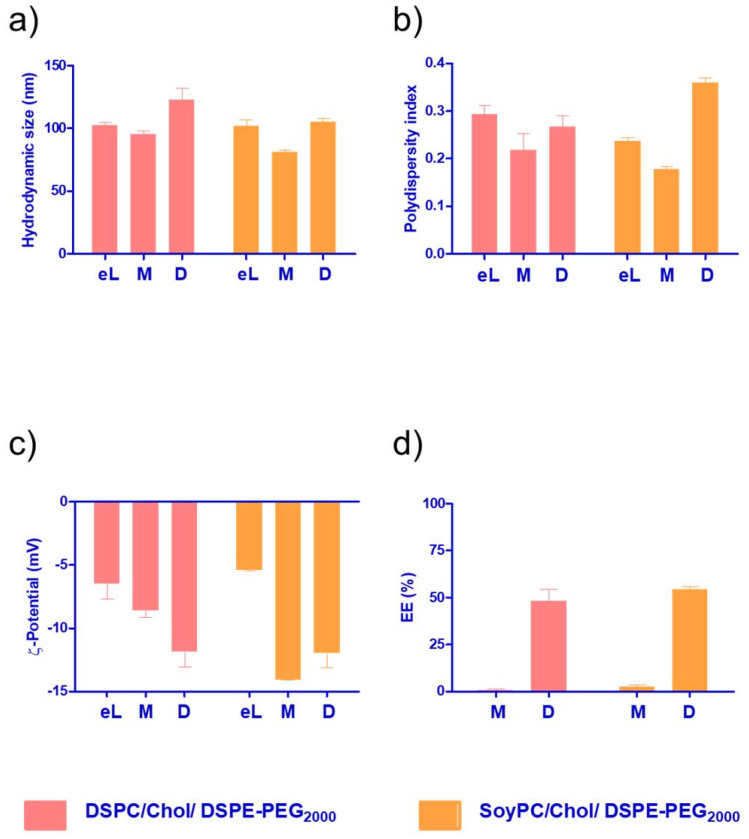
Colloidal properties and encapsulation efficiency of DSPC or SoyPC/Chol/DSPE-PEG_2000_ liposomes loaded with the monomer (M) or the dimer (D) in the bilayer of the vesicle. (a) Hydrodynamic size. (b) Polydispersity index, (c) *Z*-potential, (d) encapsulation efficiency. Data represent the mean ± SD of at least three independent measurements.

The monomeric and dimeric naphthalimides (Fig. 1a) were loaded into the liposomes *via* different procedures. Firstly, the bilayer loading was studied due to its ability to enable the encapsulation of hydrophobic drugs.^26^ The physicochemical properties of the formulations are summarised in Fig. 3. All prepared bilayer-loaded liposomes had comparable hydrodynamic diameters ranging between 80 and 120 nm. It's well described that vesicles prepared under the same conditions using saturated phospholipids tend to have a larger size than those formulated using the same unsaturated hydrocarbon chain.^27^ However, the presence of cholesterol disrupts membranes at the gel phase and condenses them at the liquid crystalline phase, modulating the properties of the bilayer, so liposomes composed of unsaturated phospholipids increase in size while those composed of saturated phospholipids decrease.^28^ Since mitonafide or the dimer analogue-loaded liposomes are expected to be administrated *via* the intravenous route, their hydrodynamic size and polydispersity index are of utmost importance to enhance the passive accumulation of the nanoparticles at the tumour site. Particles below 200 nm, like the ones obtained, can extravasate through the fenestrated vasculature of tumours, avoiding their accumulation in the liver and spleen and reducing the side toxicity of the chemotherapeutic compound.^29^

As depicted in Fig. 3b, all the formulations had a PDI value below 0.3, except for the dimer loaded in the bilayer of SoyPC liposomes, which exhibited a slight increase to 0.359. A PDI of 0.3 and below is desirable in drug delivery applications using lipid-based carriers and indicates a homogenous population of phospholipid vesicles. It is worth noting that for both the monomer and the dimer, the surface charge of the nanoparticles became more negative compared to the corresponding empty liposomes (Fig. 3c). This result suggests that both molecules are not deeply embedded in the bilayer, and parts of the molecule could be sticking out and changing the net surface charge of the particles. Interestingly, the EE% of the dimer was consistently higher (∼55%) in SoyPC or DSPC bilayers, whilst the monomer showed a poor value below 3% (Fig. 3d). Similar results were obtained for the encapsulation of the naphthalimide derivative AN169 by Parise and co-workers using a rigid phospholipid bilayer and following the same loading method.^22^ It's worth mentioning that although probe sonication is widely used for the preparation of SUVs, titanium nanoparticles may pollute the solution or sensitive amounts of lipids can be de-esterified due to the increase of temperature during the process with prolonged sonication times (≥1 h).^30^ In our case, we sonicate for a very short time, 90 seconds in total. We have analysed 3 representative samples of our formulations of mitonafide loaded in the core of SoyPC liposomes and the dimer loaded in the bilayer of SoyPC or DSPC liposomes for quantitation of the levels of Titanium using ICP-MS (ESI Table 3†). The values obtained by ICP-MS analysis were regarded as very low and only slightly elevated compared with the natural background. Therefore we can conclude that the short sonication time of our liposomes avoids the leaching of titanium nanoparticles during the preparation of the samples. If this liposomal formulation were processed for translation from bench to bedside, a necessary scale-up process is needed. Microfluidic-based manufacturing offers the opportunity to address the issue of possible contamination of titanium nanoparticles in the formulation, and de-risk the production process.^31^

**Fig. 4:**
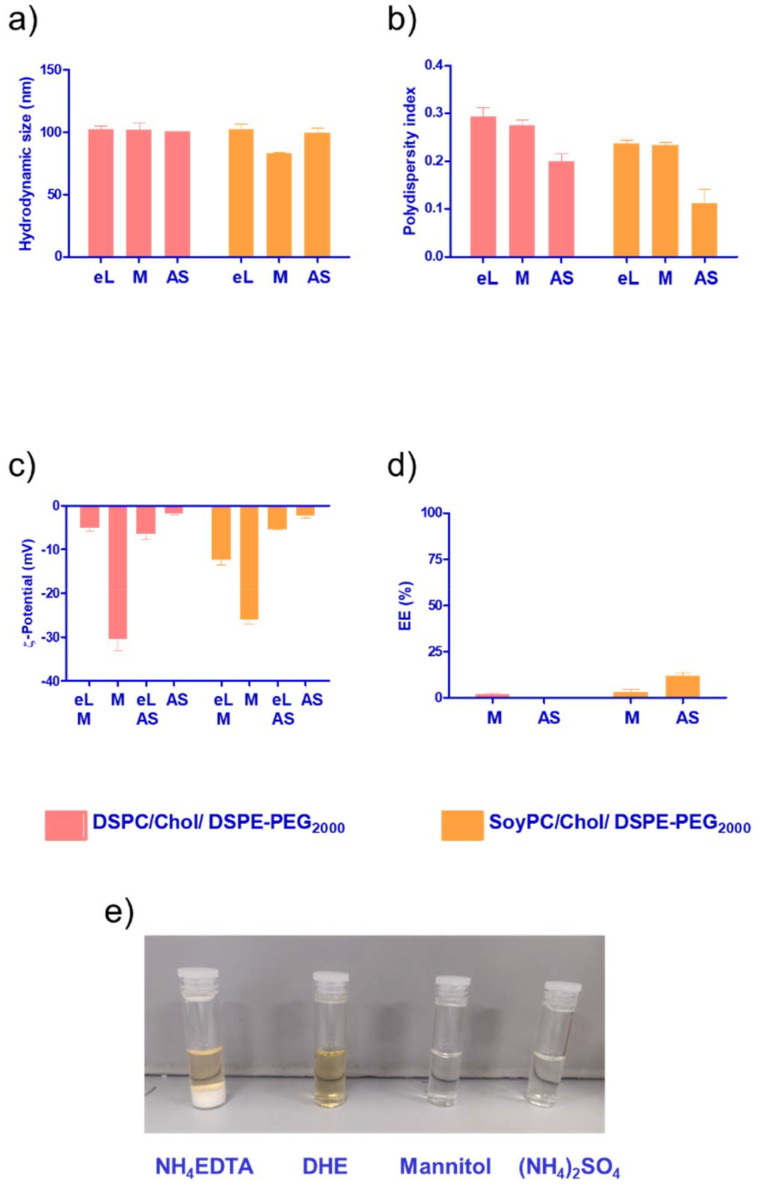
Colloidal properties and encapsulation efficiency of DSPC or SoyPC/Chol/DSPE-PEG_2000_ liposomes loaded with the monomer in the core of the vesicle using different methods (passive loading with mannitol, active loading *via* pH gradient using ammonium sulphate). (a) Hydrodynamic size. (b) Polydispersity index, (c) *Z*-potential, (d) encapsulation efficiency. (e) Solubility of the monomer in different buffers: NH_4_-EDTA (300 mM ammonium EDTA), DHE (300 mM dextrose, 20 mM HEPES and 15 mM EDTA, pH 7.4), mannitol (5% mannitol), (NH_4_)_2_SO_4_ (240 mM ammonium sulphate pH 5.4). Data represent the mean ± SD of at least three independent measurements. eL: empty liposomes, M: liposomes loaded *via* passive loading, AS: liposomes loaded *via* pH gradient.

Since the monomer had a low EE% in the bilayer of the liposomes (Fig. 3d), we decided to explore other loading methods that could improve its loading efficiency. Liposomes are an attractive drug delivery system due to their ability to incorporate their payload either in the bilayer or in the aqueous core of the nanoparticle. To study the core loading of the monomer, we selected different buffers to solubilise the molecule. As depicted in Fig. 4e, the compound formed a white precipitate in the NH_4_-EDTA buffer. In the case of DHE buffer, the monomer exhibited better solubility. However, the colour of the solution immediately changed to a dark yellow tone, suggesting a possible oxidation process. For mannitol 5% or (NH_4_)_2_SO_4_, the monomer exhibited good solubility, and no changes in colour were observed in the stock solutions.

The choice of aqueous medium enables different core loading methods, passive using a low ionic strength buffer like mannitol or active *via* pH gradient using (NH_4_)_2_SO_4_. Overall, we observed a slight reduction in the hydrodynamic size for core-loaded nanoparticles compared with the bilayer loading (size < 100 nm). It has been reported that at low ionic strength, the hydration layer around the particles is greater, resulting in premature closure of the vesicles and the formation of smaller liposomes (Fig. 4a).^32^ PDI values remained below 0.3 for all the formulations. For passively loaded liposomes using mannitol 5%, in both DSPC and SoyPC, we observed a dramatic decrease in the surface charge of the particles, suggesting that the monomer could be associated with the outer phospholipid layer in contact with the aqueous external media. In the case of liposomes loaded using pH gradient, we also observed a change in the net surface charge of the particles after loading the drug. This suggests that the monomer could stick to the membrane during the loading process and not all the molecules are dragged inside the liposomes. Interestingly, the fluidity of the membrane in this process seems to play an important role. SoyPC liposomes loaded with the monomer *via* pH gradient exhibited a 4-fold increase in encapsulation efficiency (up to 12%) compared to those with a more rigid bilayer using DSPC in the lipid composition. At pH 5.4, ammonium ions dissociate into hydrogen ions (H^+^) and ammonia (NH_3_). Hydrogen ions are charged and trapped within the intraliposomal aqueous phase. Neutral ammonia diffuses across the lipid bilayer and leaves the liposomes, further shifting the equilibrium to generate more hydrogen ions, creating an intraliposomal acidic environment. In this environment, the mitonafide amine group will have a predominantly positive charge, allowing the interaction with free sulphate ions, forming a low solubility sulphate salt that is retained in the core of the liposomes, similar to the mechanism described for the remote loading of doxorubicin described by Fritze *et al.*, 2006.^33^ Further studies should be conducted at different drug : lipid weight ratio (10 : 1 or 5 : 1) to load mitonafide *via* pH gradient in order to maximise the amount of drug loaded in the vesicles and a suitable formulation for clinical translation.

^1^H NMR analysis of the liposome particles was carried out to determine liposome stability for up to 50 days. Initial diffusion NMR measurements showed that the monomer had an observable diffusion of 9.08 × 10^−10^ M^2^ S^−1^ whilst the dimer diffused at a slightly slower rate of 6.50 × 10^−10^ M^2^ S^−1^ (ESI Fig. 4†). Solution-based NMR of the liposomes is difficult because the majority of the liposome phase is separated from the solution and thus invisible to the ^1^H spectrometer. The main exception is the DSPE-PEG component, which is assumed to extrude from the liposome surface.^34,35^ DOSY analysis of the PEG chains on the particle surface shows their diffusion varied from sample to sample. The data were treated using Stokes–Einstein equations to determine the apparent hydrodynamic radii of the PEG (its restricted mobility). Some additional peaks were visible in the liposome structures using solvent suppression – these are detailed in the ESI.† The dimer analogue was masked entirely from the spectrometer after being loaded into the DSPC bilayer but remained partially visible when loaded into the SoyPC liposomes in both the bilayer and again when the monomer was introduced into the core (see ESI†). These results agree with the ζ-potential observations for the bilayer and core-loaded liposomes exhibiting a dramatic reduction in their surface charge compared with the empty vesicles. As suggested before, during the core loading process, the monomer could stick to the membrane and not all the molecules are dragged inside the liposomes. In the case of the bilayer loading, the dimer will not be deeply embedded in the bilayer, and parts of the molecule could stick out and change the net surface charge of the particles.

**Fig. 5:**
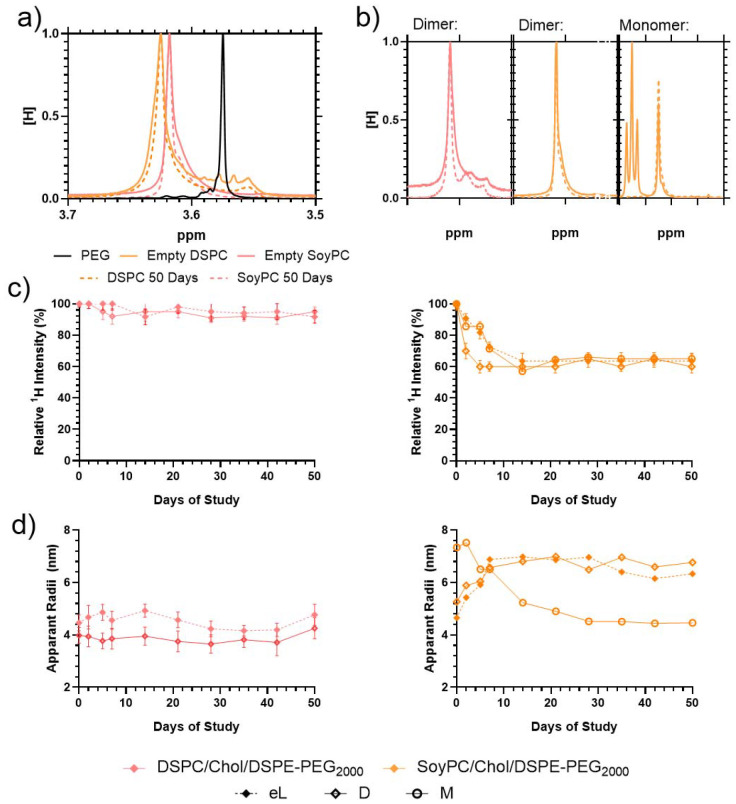
^1^H PEG (3.5–3.6 ppm) NMR analysis across storage stability test. (a) The solvent-suppressed NMR indicates the line shapes of empty liposome particles on Day 0 (solid line) and Day 50 (dashed line). (b) Solvent suppressed ^1^H NMR indicating line shapes of loaded liposome particles on Day 0 (solid line) and Day 50 (dashed line) of Dimer (left and middle) and monomer (right) systems from 3.5–3.7 ppm. (c) Relative ^1^H Intensity of PEG over sample storage (average of 3 integration comparisons). (d) Apparent hydrodynamic radii of PEG protons. Liposomes are listed as eL (empty liposomes), M (monomer loaded *via* pH gradient) and D (dimer loaded in the bilayer) systems.

Further observations were made of the samples to monitor their storage stability (Fig. 5). When incorporated into the liposome surface, the ^1^H PEG proton peak is downshifted (moved from 3.57 to ≈3.63 ppm), indicating significant broadening (Fig. 5a). Previous studies have shown that the proton peak of molecules on the liposome surfaces exhibit line broadening due to inhibited chain flexibility, so this is consistent with previous studies of equivalent systems.^34,35^ Over 50 days, the ^1^H PEG line of the liposomes, the peak width narrowed compared to the fresh liposomes (Fig. 5a and b); however, the proton intensity and apparent hydrodynamic radii of the systems differ. Over the storage period, the visible ^1^H signal from the empty DSPC liposomes retained proton intensity > 95% relative to the solvent, which is distinct from the empty SoyPC formulation, where the PEG proton signal decayed to ≈65% intensity over 7 days before remaining stable (Fig. 5c). These results agree with previous studies that have demonstrated that liposomes composed of saturated phospholipids like DSPC have rigid membranes that exhibit greater stability in the presence of serum, extended pharmacokinetics and lower burst drug leakage compared to other more flexible formulations like SoyPC.^36^

Loaded particles demonstrated similar trends, although the ^1^H intensity reduction was less rapid, taking ≈14 days to reduce an equivalent amount. DOSY measurements indicate that the apparent hydrodynamic radius (*R*_H_) of the PEG of the empty DSPC liposomes is more stable (increasing from 4.5 to 5.0 nm over the 50-day storage) whilst the SoyPC hydrodynamic radii increased over 2 weeks (rising from 4.6 to 7.0 nm) before remaining stable (Fig. 5d). Loading of the particles influenced these apparent hydrodynamic radii much more significantly. Introducing the dimer into the DSPC bilayer reduced its apparent *R*_H_ from 4.5 to 4.0 nm, which remained stable over the 50-day stability study. Conversely, introducing the dimer to the SoyPC swelled the apparent mobile PEG hydrodynamic radii to ≈5.5 nm, rapidly increasing to ≈6.5 nm over 14 days and remaining stable for the remaining storage. The insertion of this heterocyclic structure could have a similar modulating effect to cholesterol. In the ordered gel phase of stiffed membranes like DSPC, cholesterol disrupts chain packing and increases membrane fluidity, whereas, in the disordered liquid crystalline phase of the SoyPC bilayer, it condenses the membrane and decreases membrane fluidity.^37^ The addition of the monomer to the SoyPC increased its apparent *R*_H_ to ≈7 nm after loading the drug, which then decayed to ≈5 nm over 14 days of storage and remained constant for the remainder of the study. In summary, these results indicate that the DSPC liposomes show increased stability compared to the SoyPC equivalents, with the latter exhibiting some redistribution over 14 days before settling into a stable equilibrium. We also investigated the release profile of the SoyPC liposomes loaded with mitonafide in the core of the nanoparticles and the dimer analogue loaded in the bilayer of SoyPC and DSPC liposomes. Our results showed that all three liposomal formulations exhibited an initial burst release (∼40–55%) of the dimer at 37 °C in HBS compared to a lower 26% for mitonafide. This was then followed by a zero-order release kinetic for a period of 24 h, indicating a good stability of the drug carriers under these conditions (ESI Fig. 6a†). We then examined how the drug was released in presence of 50% foetal bovine serum (ESI Fig. 6b†), which resembles the biological *in vivo* environment. These results confirmed what we observed in HBS, where all formulations showed a rapid initial release of the drug. Interestingly, mitonafide loaded in the core of SoyPC liposomes exhibited a much higher release percentage up to 90% due the destabilising effect of the serum proteins. As expected, the most rigid formulation of the dimer (DSPC liposomes) showed the slowest release of the drug from the bilayer of the vesicles. In summary, the liposomes’ composition and loading method had a strong impact on the drug's release in serum, which could alter the formulations’ effectiveness *in vitro* and *in vivo*.

**Fig. 6:**
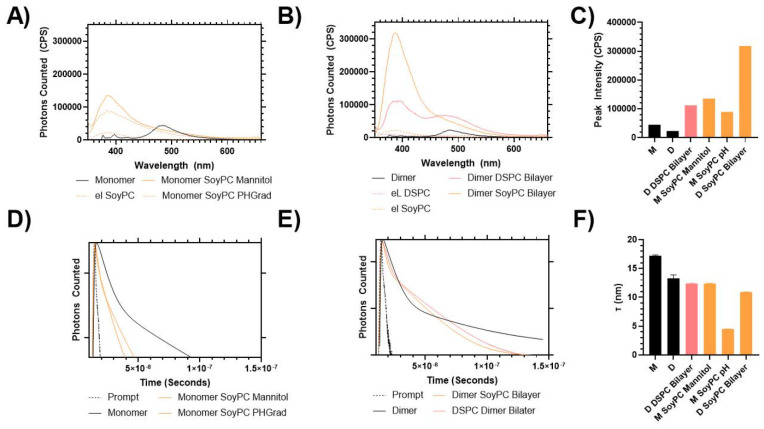
Fluorescence spectroscopy analysis of monomer (M) and dimer (D) in aqueous solution. Photoluminescence spectra following excitation at 340 nm were carried out for monomer (A) and dimer (B), to determine peak intensity (C). TCSPC measurements were also carried out on monomer (D) and dimer (E) solutions to determine the average fluorescent excited state lifetime (F). Data shows the resultant excited state lifetime determined from a dual exponential fit of the luminescent decay.

Once loaded into the liposomes, both the monomer and the dimer had an increase in fluorescence compared to the isolated aqueous solution (Fig. 6). In an aqueous solution, the monomer form demonstrates a peak emission at 490 nm, with secondary minor peaks at 400 nm.

When loaded into the SoyPC formulation, the peak intensity increased ≈1.5–2 times its initial value, but the wavelength shifted from 490 to 400 nm, indicating a colourimetric shift to more closely match the emission spectra of mitonafide in DMSO. This suggested that the molecules in the core of the liposomes have a similar arrangement to the molecules in the solution. A similar trend is observed for the dimer – however, its increase in fluorescent emission was significantly greater, particularly when loaded into the SoyPC bilayer, which provided an exponential increase in fluorescent output, which could be related to the slightly higher EE% of the flexible formulation. A time-correlated single-photon counting (TSPC) measurement was carried out on these materials, and it was found that the liposomes with reduced intensity (*i.e.* dimer DSPC bilayer, monomer SoyPC pH gradient < SoyPC mannitol) also exhibit a reduction in the excited state lifetime of the fluorescence decay. This indicates that the positioning of the mitonafide impacts its emission properties, as both monomeric and dimer forms undergo a quenching, colourimetric shift in emission when exposed to the aqueous solution. This variation in the photophysical properties of mono and bis-naphthalimides loaded in lipid-based vesicles should be considered for future applications of these molecules as bioimaging agents, chemosensors or fluorescent probes.

### *In vitro* studies

**Fig. 7:**
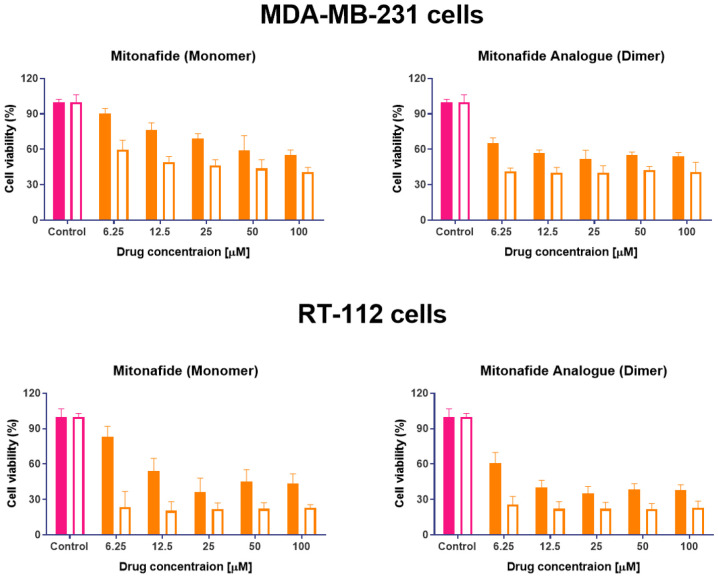
Cytotoxicity studies of mitonafide monomer and dimer in two cell lines: MDA-MB-231 breast adenocarcinoma cells and RT-112 urinary bladder carcinoma. Cells were seeded in 96-well plates (1.7 × 10^4^ cells per well), and the next day, they were incubated with both compounds over 24 (coloured bars) and 48 (white bars) hours. Cell viability was assessed using MTT assay. The results are expressed as average ± SD (*n* = 6).

This study evaluated the cytotoxic activity of both naphthalimides in MDA-MB-231 breast carcinoma and RT-112 urinary bladder transitional cell carcinoma. Cell monolayers were incubated with both compounds, and cell viability was assessed using an MTT assay after 24 and 48 hours. Results are summarised in Fig. 7. In both cell lines, the dimer exhibited lower cell viability across the entire range of concentrations. However, upon increasing drug concentration, a plateau is reached, and no further reduction in cell viability is observed, suggesting a saturation in the cellular uptake of the drugs or their binding to the DNA. These results agree with different literature reports demonstrating that synthesising two subunits of 1,8-naphthalimide connected by a linker or spacer improves DNA binding affinity and their antitumour effect.^11–14^

**Fig. 8:**
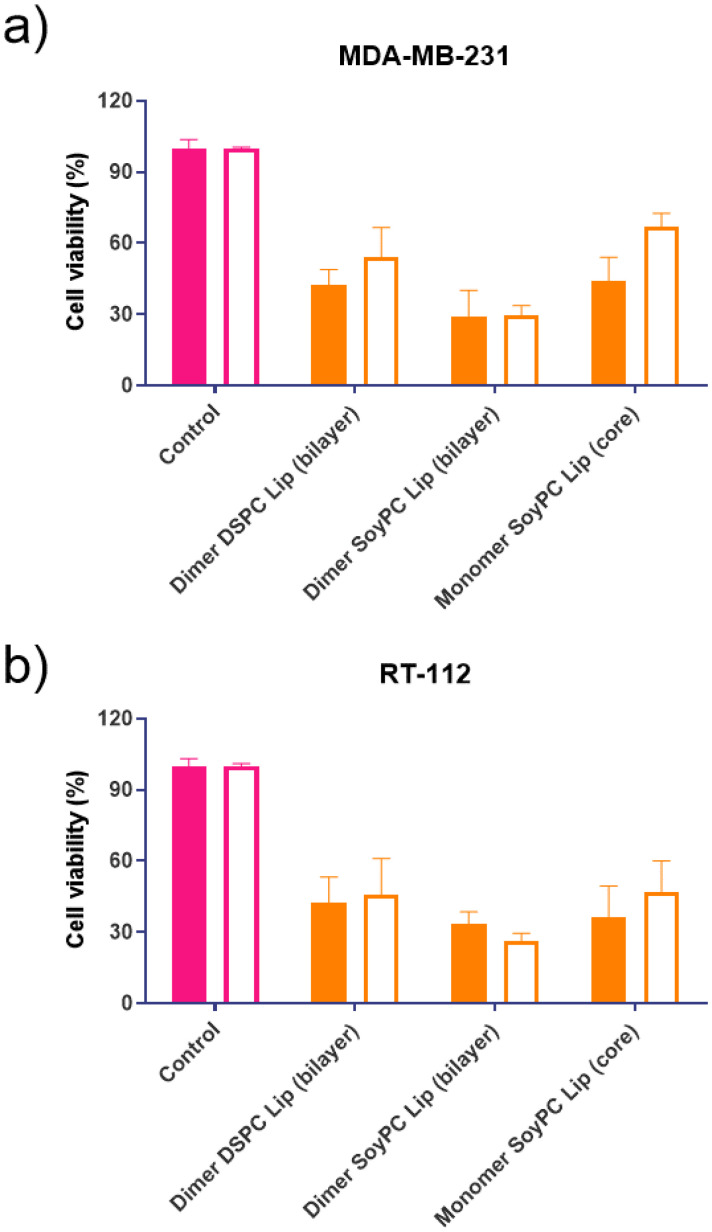
Cytotoxicity studies of different formulations loaded with the monomer or the dimer in 2 different cell lines: MDA-MB-231 breast adenocarcinoma cells (a) and RT-112 urinary bladder carcinoma (b). Cells were seeded in 96-well plates (1.7 × 10^4^ cells per well), and the next day they were incubated with different formulations over 24 (coloured bars) and 48 (white bars) hours. Cell viability was assessed using resazurin assay. The results are expressed as average ± SD (*n* = 6).

The formulation's effectiveness is primarily based on the drug release profile and how it affects their *in vitro* and *in vivo* biological activity when using drug delivery systems. To determine if the cytotoxicity of mitonafide-loaded liposomes is influenced by the physicochemical characteristics of the carrier and the loading method used, the formulations of the dimer and the monomer with the most promising results in terms of EE% were evaluated *in vitro* using MDA-MB-231 and RT-112 cells (Fig. 8). Cell monolayers were incubated with both compounds at 6.25 μM, and cell viability was assessed using a resazurin assay after 24 and 48 hours. Since the formazan granules of the MTT assay have a strong interaction with intracellular lipid droplets and lipid-based nanoparticles, we decided to use resazurin assay to assess the cytotoxicity of the formulations.^38^ As depicted in Fig. 8a, at 24 h, the dimer loaded in the bilayer of SoyPC liposomes showed quicker activity (29.3% ± 10.9) than DSPC liposomes (42.2% ± 6.63) in MDA-MB-231 cells. In contrast, the monomer loaded in the core of SoyPC liposomes showed a slower drug release, delaying the cytotoxic effect (43.9% ± 10.06). As the incubation progressed to 48 h, all three liposomal formulations showed a similar trend in their cytotoxicity. Moreover, these results were reproduced in RT-112 cells (Fig. 8b). It is worth mentioning that no antiproliferative activity was observed in cells treated with empty liposomes at lipid concentrations equivalent to the ones used with the loaded monomer and dimer (ESI Fig. 7†).

## Conclusions

This study highlights the possibility of manipulating the rigidity of the bilayer or the loading method to develop a suitable liposomal formulation of mitonafide or its dimer analogue for their therapeutic application. In this work, we have thoroughly investigated different loading methods in the bilayer or the core of the liposomes to load a mitonafide monomer or a dimer analogue. Moreover, we demonstrated that the physicochemical properties of the vesicles and the arrangement of the monomer or the dimer in the bilayer or the core of the liposomes influence their encapsulation efficacy, colloidal and structural characteristics, photoactivity and long-term stability, and how these properties could influence the *in vitro* toxicity in cancer cells. Overall, our findings could overcome the severe central nervous toxicity observed for mitonafide in clinical trials and provide a safer formulation with reduced side effects. Additionally, this research highlights the importance of structural factors that influence the photophysical properties of mono and bis-naphthalimides loaded in lipid-based vesicles so it could be relevant for a range of future applications of these molecules as bioimaging agents, chemosensors or fluorescent probes.

## Author contributions

Conceptualisation: AR, TS, KP. Data curation and formal analysis: AR, TS. Funding acquisition: AR, TS, KP. Investigation and methodology: AR, TS, RT, KP, AMD, HAAA, CCD. Project administration, resources and supervision: AR. Writing original draft, review and editing: AR, TS, KP.

## Conflicts of interest

There are no conflicts to declare.

## Supplementary Material

PM-001-D3PM00060E-s001

## References

[cit1] Berlanga J. M. C. , Brana M. F. and Roldan C. M., Patent, DE2318136A1, 1973

[cit2] Banerjee S., Veale E. B., Phelan C. M., Murphy S. A., Tocci G. M., Gillespie L. J., Frimannsson D. O., Kelly J. M., Gunnlaugsson T. (2013). Recent advances in the development of 1,8-naphthalimide based DNA targeting binders, anticancer and fluorescent cellular imaging agents. Chem. Soc. Rev..

[cit3] Braña M. F., Sanz A. M., Castellano J. M., Roldan C. M., Roldan C. (1981). Synthesis and cytostatic activity of benz de isoquinoline 1 3 diones structure activity relationships. Eur. J. Med. Chem..

[cit4] Allen S. L., Kolitz J. E., Lundberg A. S., Bennett J. M., Capizzi R. L., Budman D. R. (2010). Phase I trials of amonafide as monotherapy and in combination with cytarabine in patients with poor-risk acute myeloid leukemia. Leuk. Res..

[cit5] U.S. National , Library of Medicine, Clinicaltrials.gov, https://clinicaltrials.gov/ct2/home

[cit6] Abad A., Grávalos C., Font A., Molina F., Díaz-Puente M., Fabregat X., Benavides A., Martin M. (1996). Phase II study of Mitonafide in advanced and relapsed colorectal cancer. Invest. New Drugs.

[cit7] Rosell R., Carles J., Abad A., Ribelles N., Barnadas A., Benavides A., Martín M. (1992). Phase I study of mitonafide in 120 hours continuous infusion in non-small cell lung cancer. Invest. New Drugs.

[cit8] Llombart M., Poveda A., Forner E., Fernández-Martos C., Gaspar C., Muñoz M., Olmos T., Ruiz A., Soriano V., Benavides A. (1992). Phase I study of mitonafide in solid tumors.. Invest. New Drugs.

[cit9] Legha S. S., Ring S., Raber M., Felder T. B., Newman R. A., Krakoff I. H. (1987). Phase I clinical investigation of benzisoquinolinedione. Cancer Treat. Rep..

[cit10] Saez R., Craig J. B., Kuhn J. G., Weiss G. R., Koeller J., Phillips J., Havlin K., Harman G., Hardy J., Melink T. J. (1989). Phase I clinical investigation of amonafide.. J. Clin. Oncol..

[cit11] Braña M., Ramos A. (2001). Naphthalimides as Anticancer Agents: Synthesis and Biological Activity. Curr Med Chem Anticancer Agents.

[cit12] Kirshenbaum M. R., Chen S.-F., Behrens C. H., Papp L. M., Stafford M. M., Sun J.-H., Behrens D. L., Fredericks J. R., Polkus S. T., Sipple P., Patten A. D., Dexter D., Seitz S. P., Gross J. L. (1994). (R,R)-2,2′-[1,2-Ethanediylbis[imino(1-methyl-2,1-ethanediyl)]]-bis[5-nitro-1H-benz[de]isoquinoline-1,3-(2H)-dione] Dimethanesulfonate (DMP 840), a Novel Bis-naphthalimide with Potent Nonselective Tumoricidal Activity in Vitro. Cancer Res..

[cit13] McRipley R. J., Burns-Horwitz P. E., Czerniak P. M., Diamond R. J., Diamond M. A., Miller J. L. D., Page R. J., Dexter D. L., Chen S.-F., Sun J.-H., Behrens C. H., Seitz S. P., Gross J. L. (1994). Efficacy of DMP 840: A Novel Bis-Naphthalimide Cytotoxic Agent with Human Solid Tumor Xenograft Selectivity. Cancer Res..

[cit14] Bousquet P. F., Braña M. F., Conlon D., Fitzgerald K. M., Perron D., Cocchiaro C., Miller R., Moran M., George J., Qian X.-D., Keilhauer G., Romerdahl C. A. (1995). Preclinical Evaluation of LU79553: a Novel Bis-naphthalimide with Potent Antitumor Activity. Cancer Res..

[cit15] Wu A., Xu Y., Qian X. (2009). Novel naphthalimide–amino acid conjugates with flexible leucine moiety as side chain: Design, synthesis and potential antitumor activity. Bioorg. Med. Chem..

[cit16] Al-Jamal W. T., Kostarelos K. (2011). Liposomes: From a Clinically Established Drug Delivery System to a Nanoparticle Platform for Theranostic Nanomedicine. Acc. Chem. Res..

[cit17] Petersen G. H., Alzghari S. K., Chee W., Sankari S. S., La-Beck N. M. (2016). Meta-analysis of clinical and preclinical studies comparing the anticancer efficacy of liposomal versus conventional non-liposomal doxorubicin. J. Controlled Release.

[cit18] Gao Y.-G., Alam U., Ding A.-X., Tang Q., Tan Z.-L., Shi Y.-D., Lu Z.-L., Qian A.-R. (2018). [12]aneN3-based lipid with naphthalimide moiety for enhanced gene transfection efficiency. Bioorg. Chem..

[cit19] Carretero G. P. B., Saraiva G. K. V., Rodrigues M. A., Kiyota S., Bemquerer M. P., Chaimovich H., Cuccovia I. M. (2021). Naphthalimide-Containing BP100 Leads to Higher Model Membranes Interactions and Antimicrobial Activity. Biomolecules.

[cit20] Yang H.-Z., Zhang J., Guo Y., Pu L., Yu X.-Q. (2021). A Fluorescent Self-Reporting Vector with GSH Reduction Responsiveness for Nucleic Acid Delivery. ACS Appl. Bio Mater..

[cit21] Wang B., Zhang J., Liu Y.-H., Zhang W., Xiao Y.-P., Zhao R.-M., Yu X.-Q. (2018). A reduction-responsive liposomal nanocarrier with self-reporting ability for efficient gene delivery. J. Mater. Chem. B.

[cit22] Parise A., Milelli A., Tumiatti V., Minarini A., Neviani P., Zuccari G. (2015). Preparation, characterization and in vitro evaluation of sterically stabilized liposome containing a naphthalenediimide derivative as anticancer agent. Drug Delivery.

[cit23] Swift T., Hoskins R., Telford R., Plenderleith R., Pownall D., Rimmer S. (2017). Analysis using size exclusion chromatography of poly(N -isopropyl acrylamide) using methanol as an eluent. J. Chromatogr. A.

[cit24] Neese F., Wennmohs F., Becker U., Riplinger C. (2020). The ORCA quantum chemistry program package. J. Chem. Phys..

[cit25] Cevc G. (1991). How membrane chain-melting phase-transition temperature is affected by the lipid chain asymmetry and degree of unsaturation: an effective chain-length model. Biochemistry.

[cit26] Schwendener R. A. and Schott H., in Methods in molecular biology ed. N. J. Clifton, United States, 2010, vol. 605, pp. 129–138 10.1007/978-1-60327-360-2_8 20072877

[cit27] Guimarães Sá Correia M., Briuglia M. L., Niosi F., Lamprou D. A. (2017). Microfluidic manufacturing of phospholipid nanoparticles: Stability, encapsulation efficacy, and drug release. Int. J. Pharm..

[cit28] Ulrich A. S. (2002). Biophysical Aspects of Using Liposomes as Delivery Vehicles. Biosci. Rep..

[cit29] Blanco E., Shen H., Ferrari M. (2015). Principles of nanoparticle design for overcoming biological barriers to drug delivery. Nat. Biotechnol..

[cit30] Lombardo D., Kiselev M. A. (2022). Methods of Liposomes Preparation: Formation and Control Factors of Versatile Nanocarriers for Biomedical and Nanomedicine Application. Pharmaceutics.

[cit31] Roces C. B., Lou G., Jain N., Abraham S., Thomas A., Halbert G. W., Perrie Y (2020). Manufacturing Considerations for the Development of Lipid Nanoparticles Using Microfluidics. International Journal of Pharmaceutics.

[cit32] Sabın J., Prieto G., Ruso J. M., Hidalgo-Álvarez R., Sarmiento F. (2006). Size and stability of liposomes: A possible role of hydration and osmotic forces. Eur. Phys. J. E.

[cit33] Fritze A., Hens F., Kimpfler A., Schubert R., Peschka-Süss R. (2006). Remote loading of doxorubicin into liposomes driven by a transmembrane phosphate gradient. Biochim. Biophys. Acta, Biomembr..

[cit34] Leal C., Rögnvaldsson S., Fossheim S., Nilssen E. A., Topgaard D. (2008). Dynamic and structural aspects of PEGylated liposomes monitored by NMR. J. Colloid Interface Sci..

[cit35] Abe K., Higashi K., Watabe K., Kobayashi A., Limwikrant W., Yamamoto K., Moribe K. (2015). Effects of the PEG molecular weight of a PEG-lipid and cholesterol on PEG chain flexibility on liposome surfaces. Colloids Surf., A.

[cit36] Peetla C., Stine A., Labhasetwar V. (2009). Biophysical Interactions with Model Lipid Membranes: Applications in Drug Discovery and Drug Delivery. Mol. Pharm..

[cit37] Yeh M.-K. (2011). Hsin-I Chang and Ming-Yen Cheng, Clinical development of liposome based drugs: formulation, characterization, and therapeutic efficacy. Int. J. Nanomed..

[cit38] Angius F., Floris A. (2015). Liposomes and MTT cell viability assay: An incompatible affair. Toxicol. In Vitro.

