# Supplementary material for: Insight into the liposomal encapsulation of mono and bis-naphthalimides
Source: RSC Pharm. 2024 Mar 8;1(2):272–82. doi: 10.1039/d3pm00060e (PMC11185046; doi:10.1039/d3pm00060e)
Supplement: PM-001-D3PM00060E-s001 [file PM-001-D3PM00060E-s001.pdf]

Electronic Supplementary Material (ESI) for RSC Pharmaceutics.
This journal is © The Royal Society of Chemistry 2024

Insight into the Liposomal Encapsulation of Mono and Bis-Naphthalimides

Electronic Supplementary Information

Abdullahi Magaji Dauda,[a] Thomas Swift,[b] Richard Telford,[b] Hend AboelMagd AbdelMonem Abdelwahab,[ac] Chhanda Charan Danta,[ad] Klaus Pors,[a] and Amalia Ruiz[a*]

Chemical Synthesis

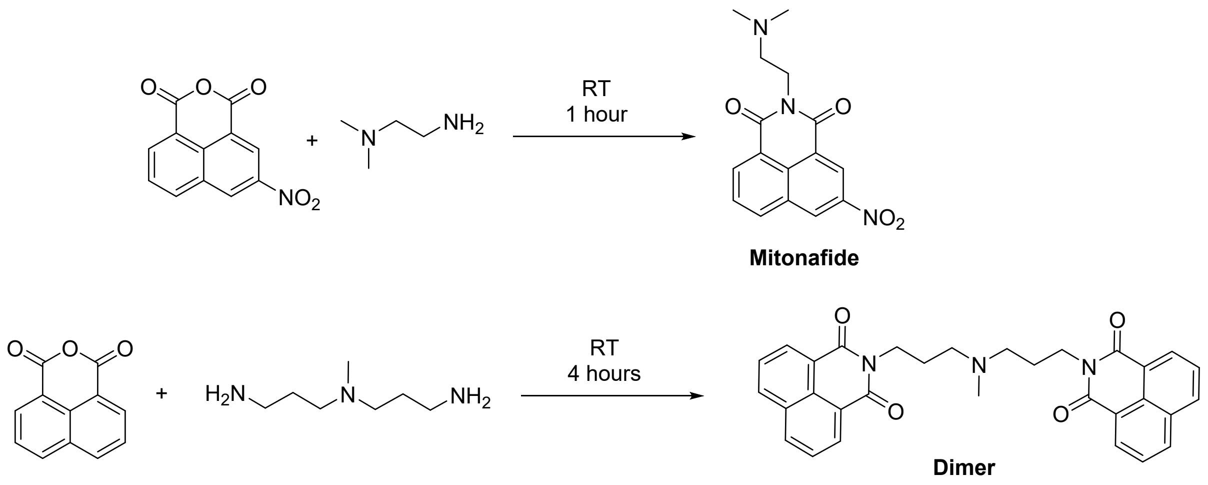

Scheme S1 – Reaction scheme demonstrating the synthesis of Mitonafide (top) and Mitonafide dimer analogue compound (bottom).

Computational Studies

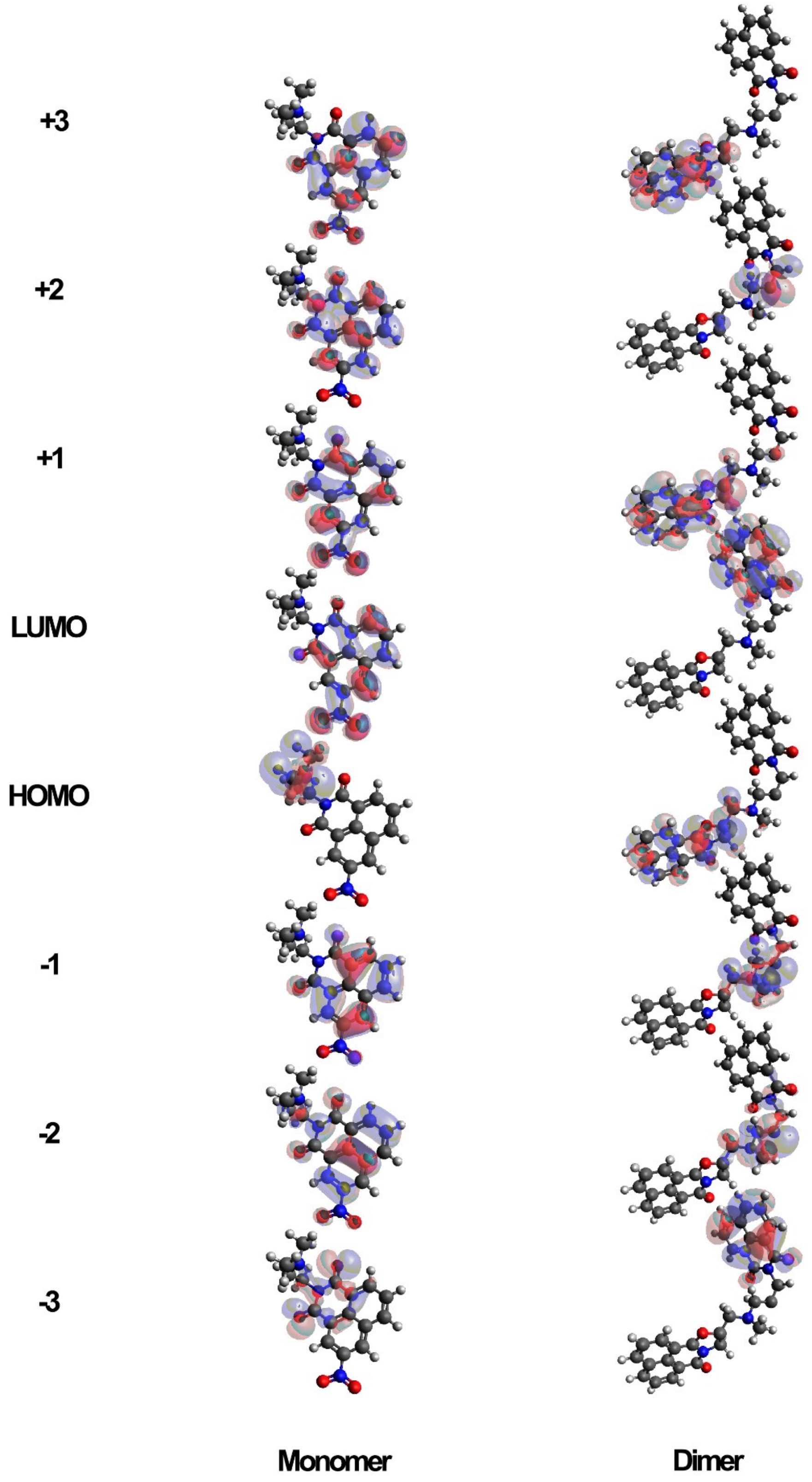

ESI Figure 1 - Calculated HOMO and LUMO structures of monomer (left) and dimer (right).

Fluorescence properties

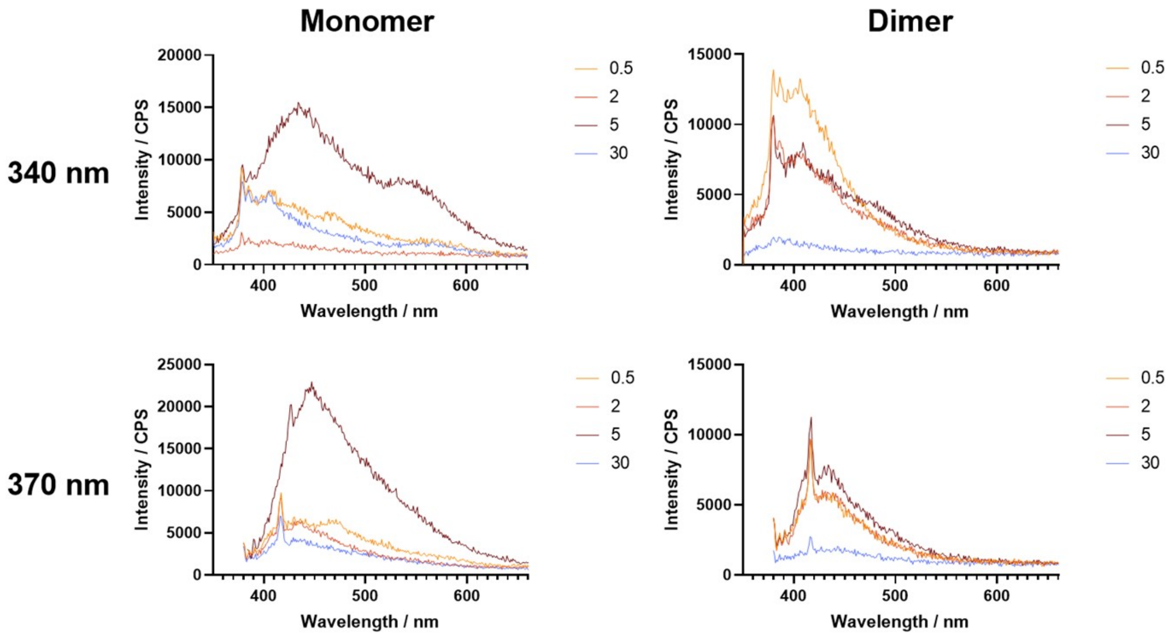

ESI Figure 2. Photoluminescence spectra of Mitonafide monomer and dimer analogue in a dilute solution of DMSO following excitation at a) 340 nm and b) 370 nm at varying molar concentrations.

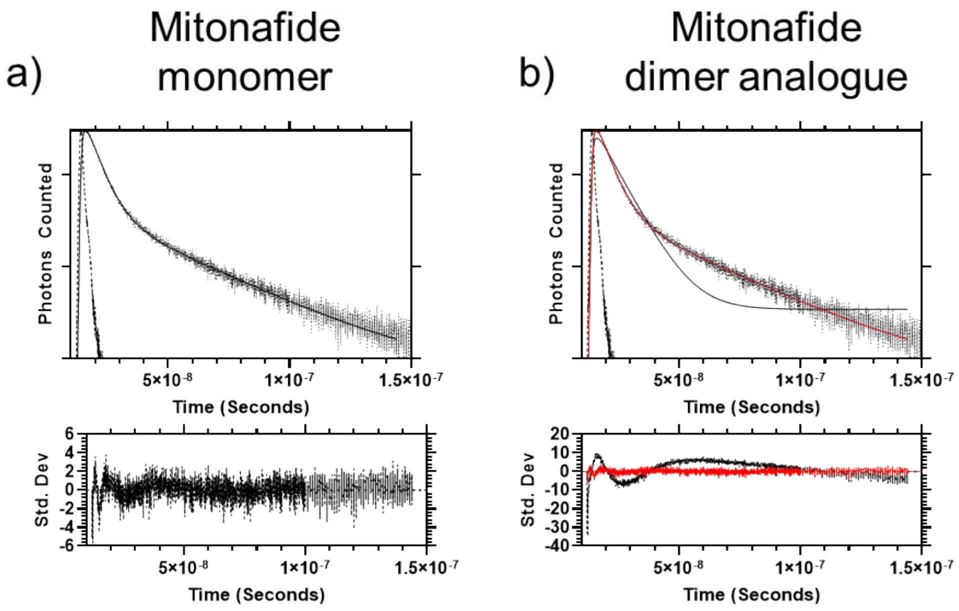

ESI Figure 3. Fluorescence decay of monomer (A) and dimer (B) in a dilute solution of DMSO. Initial fit (single exponential) is shown in black, whilst dual exponential decay is shown in red. Data in the top graph shows the scattering of the laser pulse with a silica prompt, the raw decay of the analyte and a single exponential fit. Data in the lower graph shows residual standard deviations of fit to raw data indicating the quality of the fit.

ESI Equation 1 – Equation used to determine average T from dual exponential fits.

$$T = \frac{{T_1}^2 B_2 + {T_2}^2 B_2}{T_1 B_2 + T_2 B_2}$$

ESI Table 1. Details of fluorescence decay observed to produce double exponential fits.

		**T_1**	**SD_1**	**T_2**	**SD_2**	**A**	**B_1**	**B_2**	**ChISq**	**T**	**SD**
**Free drugs**	**Monomer**	5.26	0.03	34.43	0.25	6.18	0.037	0.004	1.19	17.2	0.14
	**Dimer**	5.01	0.02	41.15	0.96	15.45	0.046	0.002	1.67	13.3	0.61
**Core loading (Passive-Mannitol)**	**Monomer-DSPC**	1.47	0.03	9.47	0.06	8.36	0.048	0.009	1.91	5.8	0.04
	**Monomer-SoyPC**	1.24	0.03	9.77	0.07	13.58	0.053	0.009	2.16	6.2	0.04
**Core loading (Active pH gradient)**	**Monomer SoyPC**	0.60	0.03	6.29	0.03	15.20	0.075	0.016	1.89	4.5	0.03
**Bilayer loading**	**Dimer DSPC**	2.74	0.03	19.92	0.11	7.75	0.037	0.007	2.68	12.4	0.06
	**Dimer SoyPC**	1.69	0.02	16.63	0.08	9.40	0.047	0.008	1.77	10.9	0.04

Diffusion NMR Measurements

**Mitonafide monomer**	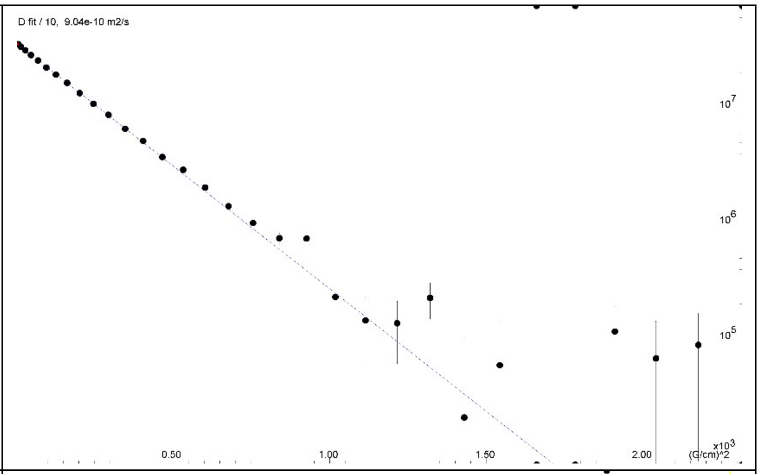
**Mitonafide dimer analogue**	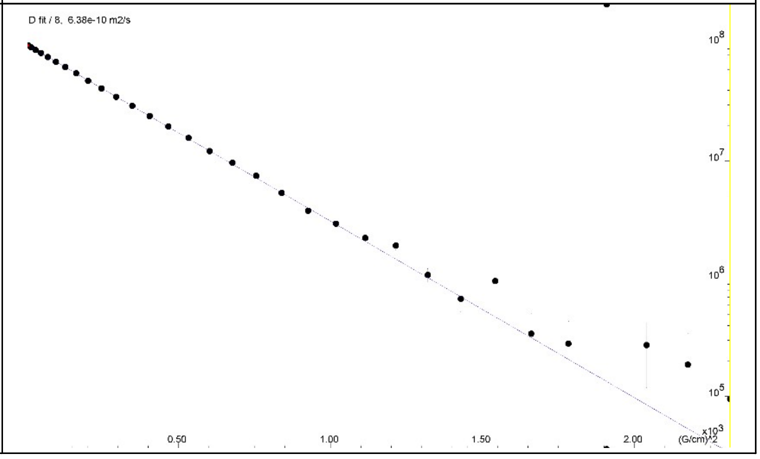

ESI Figure 4. Stejskal-Tanner plots of raw data used to determine diffusion values of Monomer and Dimer in dilute solution.

**Cholesterol**	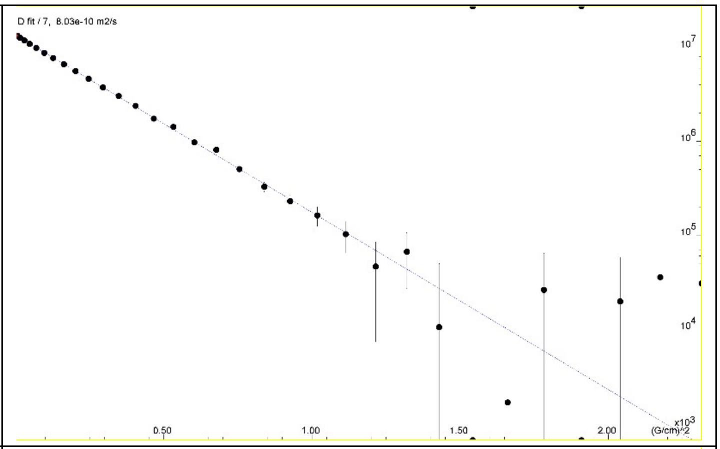
**DSPE-PEG**	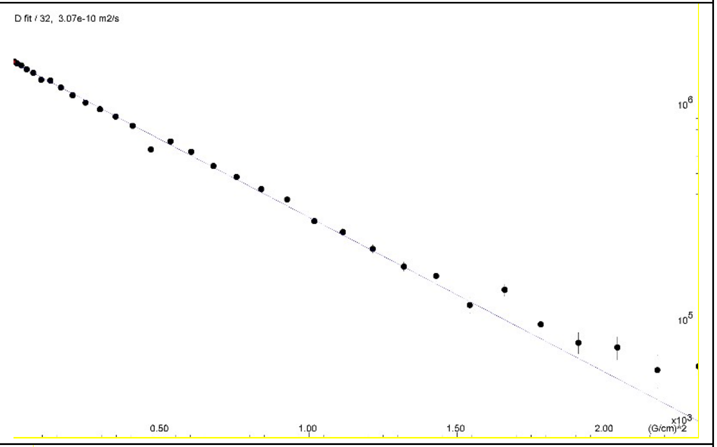
**DSPC**	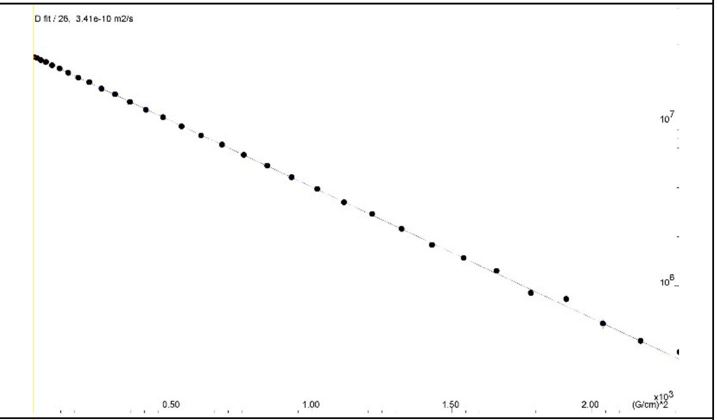
**Soy PC**	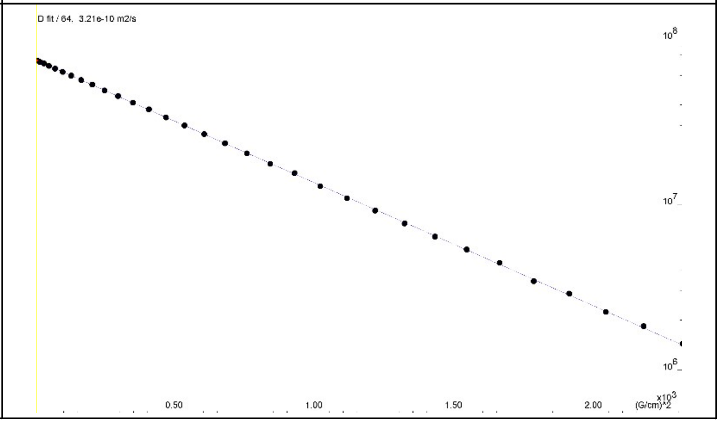

ESI Figure 5. Stejskal-Tanner plots of raw data used to determine diffusion values of individual liposome constituents in dilute solution.

a)

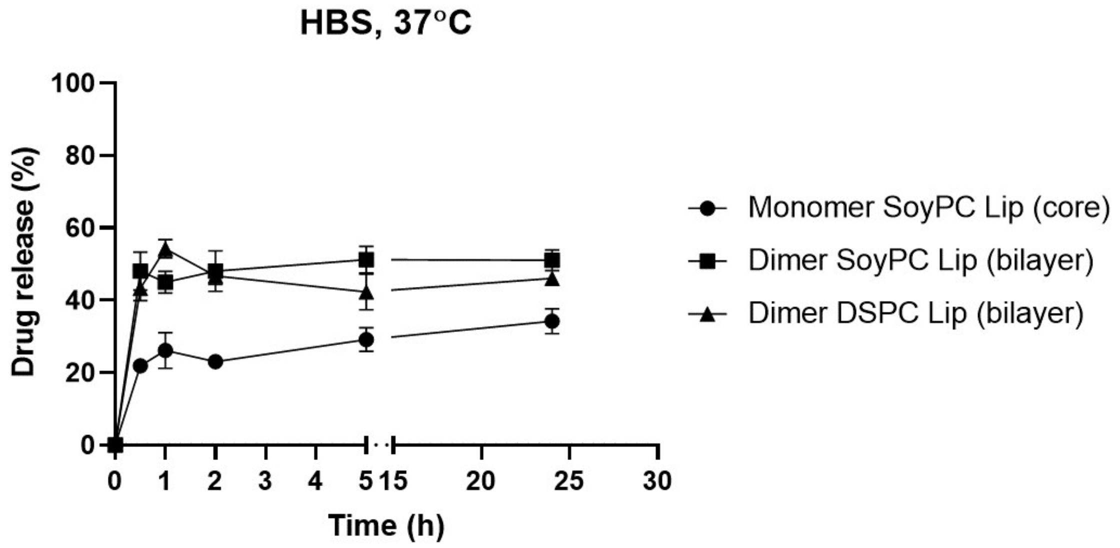

b)

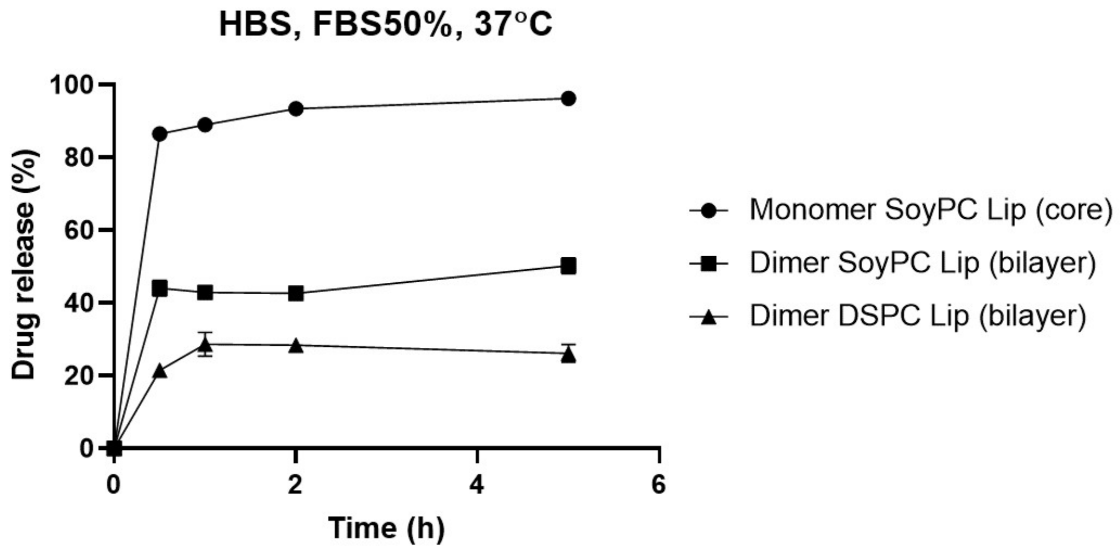

ESI Figure 6. In vitro release profile of Mitonafide or the dimer analogue loaded into different liposomes. The release profile of the liposomes incubated at 37 °C in (a) HBS buffer pH 7.4 for up to 24 h, and (b) in presence of 50% serum for up to 5 h. The release (%) was determined by HPLC, after liposome purification using a PD-10 column. Data points represent the mean ± SD of triplicate samples.

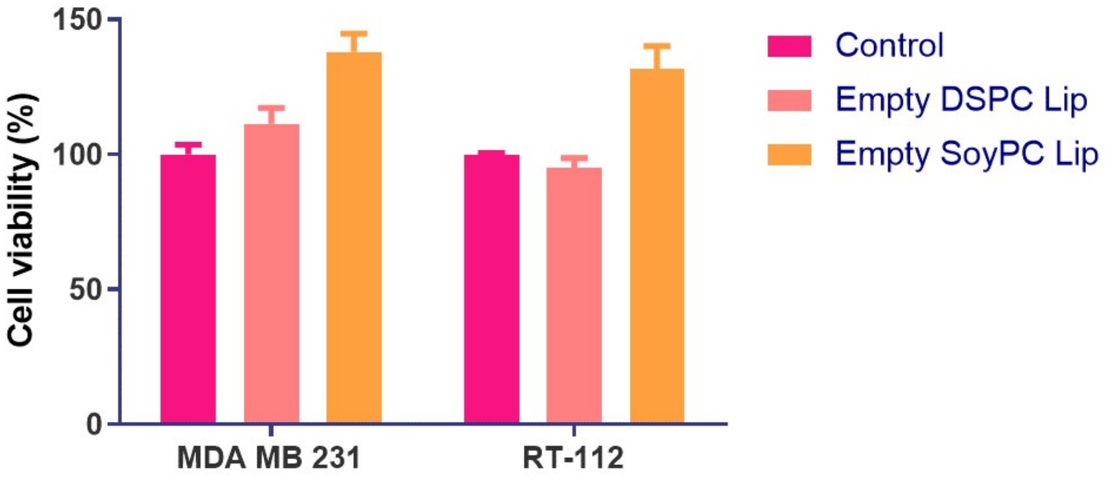

ESI Figure 7. Cytotoxicity studies of different formulations of empty liposomes in 2 different cell lines: MDA-MB-231 breast adenocarcinoma cells and RT-112 urinary bladder carcinoma. Cells were seeded in 96-well plates (1.7×10^4 cells/ well), and the next day they were incubated with different formulations at the same phospholipid concentration of the loaded liposomes over 48 hours. Cell viability was assessed using resazurin assay. The results are expressed as average ± SD (n = 6).

ESI Table 2. High-Performance Liquid Chromatography (HPLC) mobile phase gradient.

Time Gradient (minutes)	Mobile phase A	Mobile phase B	Flow (mL/min)
0:00	5.0	95.0	0.10
5:00	5.0	95.0	0.1
10:00	45.0	55.0	0.1
20:00	55.0	45.0	0.1
23:00	95.0	5.0	0.1
25:00	95.0	5.0	0.1
26:00	5.0	95.0	0.1
30:00	5.0	95.0	0.1

a)

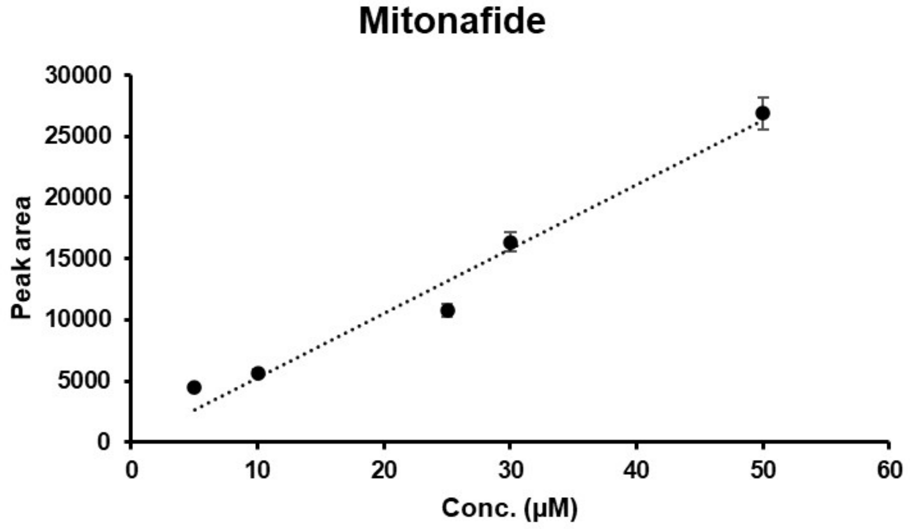

b)

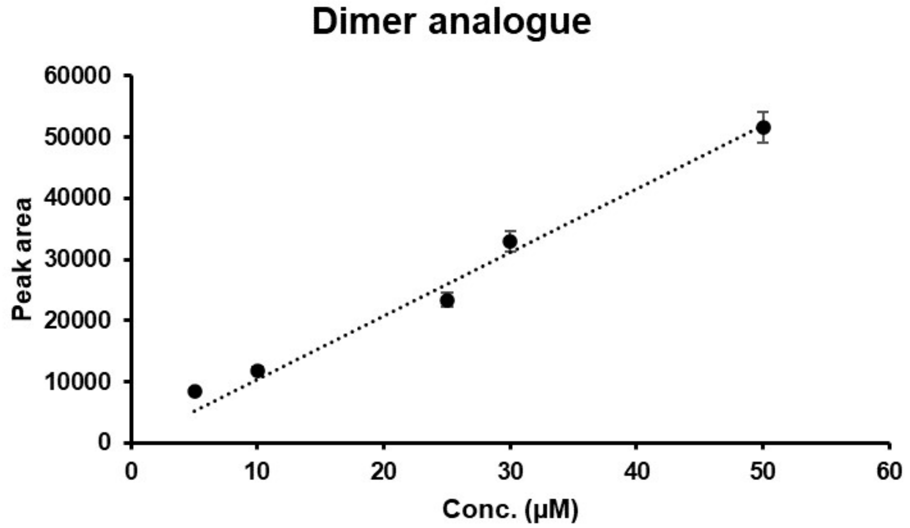

ESI Figure 8. Calibration curves of Mitonafide (a) and Dimer (b) analogue. Produced via 20 µl solution of standard, samples (n=3) at different concentrations and blank were injected into the chromatographic system. The peak was measured using absorption at 272 nm and 340 nm respectively. Linear regression analysis was used to evaluate the linearity of the calibration curve by using the least square linear regression method. Fitted linear regressions of Mitonafide had a gradient of 526.9 (R^2 = 0.991) and Dimer showed a gradient of 1039.6 (R^2 = 0.995).

ESI Table 3. Quantitation of Titanium levels in the formulations and HBS control using ICP-MS.

**Sample I.D.**	**[Titanium] / ppm**
HBS Buffer	<0.25
Mitonafide SoyPC	0.3
Dimer SoyPC	1.2
Dimer DSPC	1.5

Analytical Method for ICP-MS characterisation:

ICP-MS – Fully Quantitative for Titanium				
Instrument	Agilent 7900 ICP-MS			
Acquisition Mode:	Spectrum Analysis (multi-tune)			
Number of Masses	3			
Acquisition Parameters	Mass	Element	Time/Mass (s) No Gas Mode	Time/Mass (s) He Gas Mode
	45	Sc	N.A.	0.30
	47	Ti	N.A.	0.50
Peak Pattern	1			
Replicates	3			
Sweeps/Replicate	100			
Stabilization time / s	Tune Step 1	N.A.	Tune Step 2	20
Peristaltic Pump Program (Sample introduction + probe rinse)	Uptake Speed / rps	Uptake Time / s	Stabilization time / s	
Before Acquisition	0.50	35	30	
Probe Rinse	0.30	10	N.A.	
Peristaltic Pump Program (Rinse)	Rinse speed / rps	Rinse on rinse vial /s	Rinse on rinse port / s	
Rinse Vial 1 (5% HNO_3/0.5% HCl/0.1% HF)	0.40	45	0	
Rinse Vial 1 (2% HNO_3/0.5% HCl/0.1% HF)	0.40	45	0	

Sample Preparation:

- Approximately 200 mg of each sample was accurately weighed, in duplicate, into microwave digestion tubes followed by 8.0ml of concentrated, trace-metal grade nitric acid and 2.0 ml of concentrated, trace-metal grade hydrofluoric acid. The tubes were then sealed and digested using the parameters shown below:

Microwave Digestion	
Instrument	Milestone Ethos EZ
Rotor + Vessels	SK12 Rotor + TFM Vessels
Sample Mass / mg	200
Digestion Solution	8.0 ml of concentrated, trace-metal grade nitric acid and 2.0 ml of concentrated, trace-metal grade hydrofluoric acid
Temperature Program	20 mins to 200°C then hold for 20 mins.
Microwave Power / W	1200
Safety Temperature (T2) / ^{O}C	130

- The resulting solutions were allowed to cool to room temperature then quantitatively transferred to 120 ml polypropylene screw-cap jars using approximately 50 ml of ultra-pure water.

- 1000 µl of the 2 ppm scandium internal standard stock was pipetted into each jar, which were then made to the 100 ml graduation with ultra-pure water and well mixed.
- An aliquot of each of the solutions were transferred to ICP-MS sample tubes for analysis.
